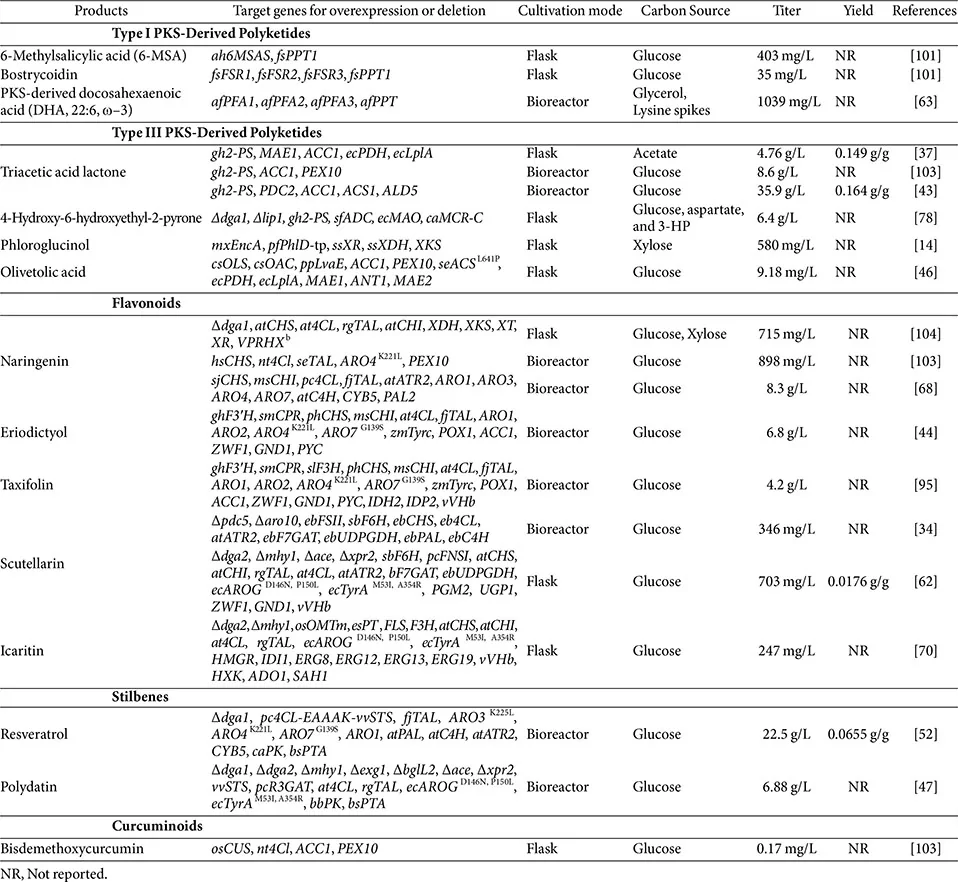# Toward Complex Polyketide Biosynthesis in *Yarrowia lipolytica* via Metabolic Reprogramming

**DOI:** 10.4014/jmb.2606.06035

**Published:** 2026-08-21

**Authors:** Kian Ghaempanah, So-Hee Son, Ju Young Lee

**Affiliations:** 1 Department of Chemical and Biomolecular Engineering (BK21 four), Korea Advanced Institute of Science and Technology (KAIST), Daejeon 34141, Republic of Korea; 2 Graduate School of Engineering Biology, Korea Advanced Institute of Science and Technology (KAIST), Daejeon 34141, Republic of Korea; 3 Department of Biological Sciences, Korea Advanced Institute of Science and Technology (KAIST), Daejeon 34141, Republic of Korea; 4 KI for the Bioinnovation, Korea Advanced Institute of Science and Technology (KAIST), Daejeon 34141, Republic of Korea

**Keywords:** Polyketide, Natural product, Microbial cell factories, Metabolic engineering, *Yarrowia lipolytica*

## Abstract

Polyketides are among the most structurally diverse and therapeutically important classes of natural products, serving as antibiotics, anticancer agents, agrochemicals, and industrial pigments. Their structural complexity and limited natural availability have driven the development of microbial biosynthetic platforms as scalable and sustainable alternatives to traditional extraction or chemical synthesis. *Yarrowia lipolytica*, a non-conventional and metabolically versatile yeast, has emerged as a promising alternative chassis for polyketide biomanufacturing, owing to its streamlined central metabolism, high acetyl-CoA availability, and exceptional physiological robustness. Recent advances in metabolic engineering and synthetic biology have enabled extensive rewiring of *Y. lipolytica* metabolism to support the high-yield production of complex, high-value polyketides. This review summarizes state-of-the-art strategies, from classical metabolic rewiring to emerging approaches such as organelle engineering and subcellular compartmentalization. We further highlight representative case studies of polyketide biosynthesis in *Y. lipolytica*, critically assess current limitations, and explore future directions to establish this organism as a programmable, industrially viable platform for polyketide production.

## Introduction

Polyketides are an extremely diverse class of natural compounds, widely used as antibiotics (*e.g.*, erythromycin), cardiovascular statins (*e.g.*, lovastatin), anticancer agents (*e.g.*, doxorubicin), immunosuppressants (*e.g.*, rapamycin), insecticides (*e.g.*, spinosad), and natural dyes (*e.g.*, carminic acid) [1]. Polyketides are biosynthesized via iterative Claisen condensations of acyl-CoA building blocks—most commonly malonyl-CoA, but also acetyl-CoA, propionyl-CoA, methylmalonyl-CoA, and hexanoyl-CoA—catalyzed by polyketide synthases (PKSs). PKSs are classified into three major types based on their structural and mechanistic characteristics [2-4]. Type I PKSs are large, multimodular enzymes, with each module catalyzing a single round of chain extension. Type II PKSs consist of discrete enzymes with at least three core components, collectively known as the minimal PKS, which catalyze the formation of aromatic carbon scaffolds. Type III PKSs are homodimeric enzymes that directly utilize acyl-CoA substrates to synthesize aromatic polyketide backbones without the involvement of acyl carrier proteins (ACPs) [2, 5, 6]. Across all PKS types, malonyl-CoA functions as the principal extender unit, although other acyl-CoA precursors, such as acetyl-CoA, propionyl-CoA, hexanoyl-CoA, and *p*-coumaroyl-CoA, are also employed depending on the PKS class and the end product [7].

Despite their pharmaceutical and industrial significance, the biosynthetic logic underlying polyketide assembly remained largely elusive for decades. In particular, the structure and functional programming of PKSs, which govern the assembly of diverse polyketide scaffolds, were poorly understood [8]. Similarly, traditional chemical synthesis is hindered by the inherent structural complexity of polyketides [9, 10]. Given these challenges, microbial biosynthesis platforms have emerged as a sustainable alternative for scalable polyketide production [7, 11]. However, conventional microbial hosts such as *Escherichia coli* and *Saccharomyces cerevisiae* face several intrinsic limitations, including inefficient expression of heterologous PKSs, restricted availability of essential precursors, and suboptimal tolerance to industrial fermentation stress. In particular, these conventional hosts suffer from low intracellular pools of malonyl-CoA, a key extender unit in polyketide biosynthesis [7, 12]. For instance, *E. coli* often exhibits limited compatibility with the functional expression of complex eukaryotic PKSs and membrane-associated tailoring enzymes, partly due to the absence of eukaryotic endomembrane systems and organelle-based compartmentalization [13]. Meanwhile, *S. cerevisiae* shows reduced tolerance to several polyketide pathway intermediates, such as malonic acid and phloroglucinol, which can accumulate during polyketide biosynthesis and exert cytotoxic effects that impair cell growth [14]. These limitations have motivated the exploration of alternative chassis organisms with improved biosynthetic flexibility, increased precursor availability, and greater physiological robustness to support efficient polyketide production.

Among alternative microbial hosts, *Yarrowia lipolytica* has attracted growing attention as a potent chassis due to its favorable metabolic properties, physiological traits, and an expanding genetic toolbox, including CRISPR-based genome editing systems (Table 1). This oleaginous yeast features a streamlined central carbon metabolism with naturally high flux through acetyl-CoA and malonyl-CoA pathways—two key precursors for polyketide assembly [15-17]. In addition to this metabolic strength, *Y. lipolytica* possesses remarkable feedstock flexibility, capable of metabolizing a broad spectrum of carbon sources, including hydrophilic substrates such as organic acids, sugars, and glycerol, as well as hydrophobic feedstocks such as alkanes and fatty acids [18, 19]. This metabolic flexibility is further enhanced by its robustness under industrially relevant conditions, including low pH, osmotic and oxidative stress, high aeration, and toxic intermediates [20, 21]. Notably, engineered *Y. lipolytica* has been shown to accumulate over 60 g/L of malonic acid, highlighting its remarkable tolerance to malonic acid precursors and its metabolic flux capacity relative to conventional yeast hosts [22].

In parallel, recent advances in genetic, regulatory, and computational engineering tools have further empowered *Y. lipolytica* as a versatile industrial chassis for diverse biotechnological applications [21, 23]. Key advances include CRISPR/Cas-based genome editing, modular expression systems, and metabolic modeling frameworks, which have enabled the efficient biosynthesis of diverse acetyl-CoA- and malonyl-CoA-derived products, such as terpenoids, organic acids, sugar alcohols, and various lipids and lipid derivatives [24-29]. In addition to efficient genome editing, CRISPR interference (CRISPRi) and CRISPR activation (CRISPRa) have emerged as promising tools for fine-tuning the expression of essential genes without permanent gene disruption, enabling multiplex metabolic pathway rewiring. These systems could enable multiplexed metabolic pathway rewiring by attenuating essential competing pathways, such as core lipid biosynthesis, while simultaneously activating precursor-supplying pathways. Furthermore, recent large-fragment DNA integration strategies facilitate the stable genomic integration of multi-gene pathways, providing a practical route for expressing large and complex PKS gene clusters [30, 31].

Recent reviews have comprehensively summarized the expanding synthetic biology toolbox of *Y. lipolytica* and its broad biotechnological applications in the production of diverse natural products and value-added compounds [32, 33]. In contrast, this review provides a distinct, integrated engineering framework for complex polyketide biosynthesis, linking recent advances in metabolic and cellular engineering with current challenges and future opportunities. Accordingly, this review summarizes recent progress in engineering *Y. lipolytica* as a microbial chassis for polyketide biosynthesis, covering both classical metabolic engineering strategies—such as enhancing malonyl-CoA flux, cofactor balancing, and pathway optimization—as well as emerging synthetic biology approaches, including organelle-based pathway compartmentalization.

## Metabolic Engineering Strategies for Efficient Polyketide Biosynthesis

### Enhancing Malonyl-CoA Availability

**Fig. 1:**
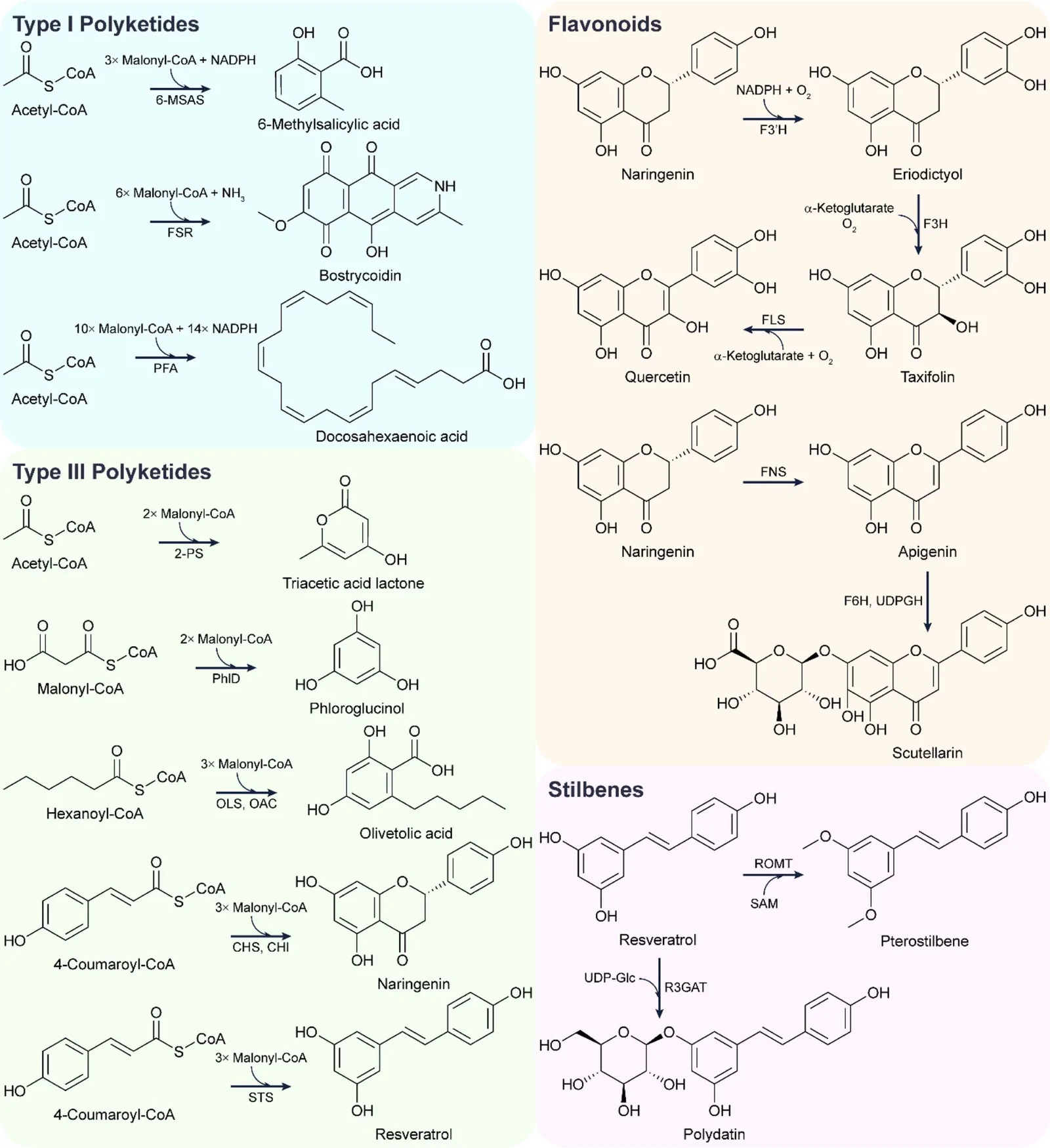
Polyketides and polyketide-derived natural products in *Yarrowia lipolytica*. Representative compounds are categorized according to the type of polyketide synthase (PKS) involved in their biosynthesis and are grouped in color-coded boxes to distinguish major classes: Type I polyketides (blue), Type III polyketides (green), flavonoids (orange), and stilbenes (pink). Type I PKSs produce 6-methylsalicylic acid (6-MSA), bostrycoidin, and docosahexaenoic acid [34]. Type III PKSs produce triacetic acid lactone (TAL), phloroglucinol, olivetolic acid, naringenin, and resveratrol. Each pathway illustrates the respective starter unit (*e.g.*, acetyl-CoA, malonyl-CoA, hexanoyl-CoA, or 4-coumaroyl-CoA), the number of incorporated malonyl-CoA extender units, and the required cofactors. Downstream modifications of naringenin and resveratrol generate structurally diverse flavonoid and stilbenoid derivatives. CoA, coenzyme A; SAM, *S*-adenosylmethionine; UDP-Glc, uridine diphosphate glucose; 6-MSAS, 6-methylsalicylic acid synthase; FSR, fusarubin synthase; PFA, polyunsaturated fatty acid synthase; 2-PS, 2-pyrone synthase; PhlD, phloroglucinol synthase; OLS, olivetol synthase; OAC, olivetolic acid cyclase; CHS, chalcone synthase; STS, stilbene synthase; F3'H, flavonoid 3'-hydroxylase; F3H, flavanone 3-hydroxylase; FLS, flavonol synthase; FNS, flavone synthase; F6H, flavone 6-hydroxylase; UDPGH, UDP-glucuronosyltransferase; ROMT, resveratrol O-methyltransferase; R3GAT, resveratrol 3-*O*-glucosyltransferase.

Malonyl-CoA serves as the primary extender unit in polyketide biosynthesis and is widely recognized as a major metabolic bottleneck in microbial polyketide production. The intracellular demand for malonyl-CoA varies depending on the specific polyketide: for instance, the synthesis of naringenin, a representative type III polyketide and a flavonoid precursor, consumes three malonyl-CoA molecules, while more complex molecules such as docosahexaenoic acid [34], a type I polyketide, consume up to ten [12] (Fig. 1). To address this limitation, metabolic engineering strategies have focused on three key aspects: (i) enhancing malonyl-CoA synthesis by modulating endogenous biosynthetic enzymes such as acetyl-CoA carboxylase (ACC), (ii) increasing the supply of its immediate precursor, acetyl-CoA, and (iii) alleviating metabolic competition from lipid biosynthetic pathways that directly compete with PKSs for the malonyl-CoA pool.

Malonyl-CoA is synthesized by ACC, a biotin-dependent enzyme that catalyzes the ATP-dependent carboxylation of acetyl-CoA [35]. As the sole enzymatic route to malonyl-CoA, ACC plays a central role in controlling its intracellular pool. Overexpression of the native *ACC1* gene in *Y. lipolytica* has been shown to significantly elevate malonyl-CoA levels [36-38]. However, native Acc1 activity is negatively regulated through Snf1-mediated phosphorylation, which inhibits its catalytic function [39, 40]. To mitigate this regulatory constraint, phosphorylation sites have been mutated (Acc1^S667A/S1178A^ variant), preventing Snf1-dependent inactivation and thereby maintaining constitutive enzyme activity, ultimately increasing the malonyl-CoA pool [22].

Since acetyl-CoA is the direct precursor of malonyl-CoA, maintaining a sufficient cytosolic acetyl-CoA pool is essential. In *Y. lipolytica*, acetyl-CoA is primarily derived from mitochondrial citrate export and its subsequent conversion by ATP citrate lyase (ACL). Accordingly, enhancing ACL activity has been used to increase acetyl-CoA availability for malonyl-CoA-derived product biosynthesis. For example, heterologous expression of a high-affinity ACL from *Mus musculus* increased lipid content from 7.3% to 23.1% of dry cell weight [41]. Similarly, overexpression of the native ACLs (*ACL1* and *ACL2*) increased docosapentaenoic acid (DPA) production by 41%, reaching a final titer of 634.0 mg/L [42].

Together with boosting acetyl-CoA synthesis, recycling carbon through peroxisomal β-oxidation is a well-established strategy for regenerating acetyl-CoA from stored lipids. In *Y. lipolytica*, overexpression of *PEX10*, a key regulator of peroxisome biogenesis, increased triacetic acid lactone (TAL) production by 22% through enhanced acetyl-CoA recycling from lipid degradation [43]. Similarly, overexpression of *POX1*, a rate-limiting enzyme in fatty acid β-oxidation, improved eriodictyol production by 18% by promoting the conversion of stored lipids into acetyl-CoA. These studies suggest that coordinated regulation of lipid turnover and β-oxidation is an important strategy for supporting the biosynthesis of malonyl-CoA-derived products [44].

In another study, heterologous acetyl-CoA generation routes—beyond optimizing native pathways—have also been implemented to enable orthogonal metabolic rewiring, as non-native enzymes are often less susceptible to endogenous regulatory mechanisms. A representative example is the incorporation of the *S. cerevisiae* carnitine acetyltransferase (Cat2) into *Y. lipolytica*, which facilitates acetyl-CoA shuttling between mitochondria and the cytosol and improves cytosolic acetyl-CoA availability for lipid biosynthesis. This strategy resulted in a 3.1-fold increase in lipid productivity compared with the parental strain [45]. Likewise, reconstructing the *E. coli* pyruvate dehydrogenase complex (PDH) together with co-expression of the lipoylating enzyme LplA in *Y. lipolytica* enhanced TAL production by 37% [37]. This heterologous PDH-based acetyl-CoA-generating module has also been employed in olivetolic acid-producing strains to further improve precursor availability for type III polyketide biosynthesis [46]. Moreover, redirecting carbon flux through the phosphoketolase (PK) shunt provides an alternative route to boost cytosolic acetyl-CoA availability while simultaneously enhancing the supply of erythrose-4-phosphate (E4P). Introduction of a heterologous phosphoketolase–phosphotransacetylase pathway (PK–PTA) module in *Y. lipolytica* increased resveratrol production by 21.8%, demonstrating the utility of carbon flux rewiring for polyketide biosynthesis [47]. Together, these metabolic engineering strategies—including enhanced ACC activity, expanded acetyl-CoA synthesis, and orthogonal carbon flux rewiring—have significantly improved malonyl-CoA availability, thereby enabling more efficient polyketide biosynthesis in *Y. lipolytica*.

### Redirecting Malonyl-CoA from Competing Endogenous Pathways

The oleaginous nature of *Y. lipolytica* creates a unique metabolic landscape for polyketide biosynthesis, offering both advantages and potential trade-offs. While its metabolic architecture favors the generation of acetyl-CoA and malonyl-CoA, these critical precursors are also strongly diverted into native lipid biosynthetic pathways—particularly fatty acid and triacylglycerol synthesis [48-50]. Indeed, a substantial portion of malonyl-CoA is consumed by endogenous lipid biosynthesis, which serves as a major competing sink for this critical precursor and significantly limits its availability to heterologous PKS pathways. To cope with this challenge, metabolic engineering strategies have focused on reducing flux into lipid biosynthesis while maintaining the minimal lipid production necessary for membrane integrity and cell viability.

One commonly explored approach involves repressing fatty acid synthase (FAS), the primary consumer of malonyl-CoA in de novo fatty acid synthesis. As a proof of concept, chemical inhibition of FAS using cerulenin, a potent fungal FAS inhibitor, increased TAL production by 2.7-fold by redirecting acetyl-CoA and malonyl-CoA away from fatty acid biosynthesis and toward polyketide production [51]. Another effective strategy involves modifying the pathways responsible for lipid storage. In *Y. lipolytica*, TAGs, a major class of storage lipids in eukaryotes, are synthesized predominantly by the acyltransferases (Dga1 and Dga2) and phospholipid:diacylglycerol acyltransferase (Lro1). Deletion of *DGA1* increased resveratrol production by 30% [52], while a *DGA1*/*DGA2* double knockout enhanced malonic acid production by 63% and simultaneously reduced total lipid accumulation by 36% [22].

However, permanent disruption of these core lipid biosynthetic pathways can introduce substantial metabolic trade-offs by compromising membrane homeostasis, stress tolerance, and overall cellular fitness in an oleaginous yeast. Dynamic regulatory strategies offer an attractive alternative by enabling temporal control of metabolic flux. For example, growth phase-dependent or chemically inducible promoters can decouple biomass accumulation from product synthesis, allowing competing lipid pathways to function during cell growth while being selectively attenuated during the production phase. Such temporal regulation can reduce the physiological cost of permanent pathway disruption while improving carbon partitioning toward complex polyketide biosynthesis [53].

Phospholipid metabolism also contributes to intracellular lipid partitioning and modulates the balance between membrane lipid synthesis and storage lipid accumulation [54]. For instance, deletion of *OPI3*, involved in phosphatidylcholine biosynthesis, increased TAG accumulation by 43%. Furthermore, when combined with the expression of an attenuated *CDS1^P367S^* variant—encoding a phosphatidate cytidylyltransferase involved in CDP-diacylglycerol synthesis—phospholipid biosynthesis was further reduced and improved TAG production by an additional 23% [55]. Moreover, sterol biosynthesis consumes acetyl-CoA and competes with polyketide production for precursor availability [56]. In *Aspergillus terreus*, disruption of the sterol regulatory system by deleting SCAP or SREBP increased lovastatin production by 5.1-fold and 2.6-fold, respectively [57, 58]. Although such strategies have not yet been applied in *Y. lipolytica*, they represent a promising avenue for future metabolic engineering.

Finally, non-lipid carbon-storage pathways can further constrain the availability of acetyl-CoA and malonyl-CoA. For instance, carbon allocation toward glycogen synthesis can indirectly compete with acetyl-CoA- and malonyl-CoA-dependent biosynthetic pathways. Deletion of the glycogen synthase gene *GSY1* abolished glycogen accumulation and increased TAG content by up to 17%, indicating that glycogen synthesis competes with lipid storage for carbon allocation [59]. By systematically reducing flux through these diverse competing endogenous pathways, these strategies work synergistically with precursor-enhancing approaches to enable more effective allocation of malonyl-CoA to heterologous biosynthetic pathways, ultimately improving the efficiency and titer of polyketide production in *Y. lipolytica*.

### Engineering Cofactor Balance

Polyketide biosynthesis is a highly energy- and redox-demanding process that requires a continuous supply of essential cofactors such as NADPH and coenzyme A (CoA). These cofactors play essential roles in maintaining metabolic flux, supporting enzymatic activities of PKSs, and enabling precursor biosynthesis, including the formation of malonyl-CoA. Accordingly, beyond precursor and flux optimization, engineering cofactor availability has emerged as an additional lever for enhancing polyketide production.

As a central reducing cofactor, NADPH is indispensable for anabolic pathways, including fatty acid and polyketide biosynthesis, while also supporting the antioxidant defense systems that maintain cellular redox homeostasis under environmental and metabolic stress [60, 61]. In *Y. lipolytica*, cytosolic NADPH is predominantly generated through the oxidative pentose phosphate pathway. Co-overexpression of *ZWF1* and *GND1*—which encode glucose-6-phosphate dehydrogenase and 6-phosphogluconate dehydrogenase, the first two enzymes of this pathway, respectively—enhanced NADPH regeneration, thereby increasing the production of the flavonoid-type polyketide scutellarin by approximately 28% [62]. In parallel with cofactor engineering, systematic optimization of the scutellarin biosynthetic pathway through enzyme selection and pathway balancing enabled de novo production of 346 mg/L scutellarin in a 1.3-L bioreactor, further demonstrating the potential of *Y. lipolytica* for complex flavonoid biosynthesis [34].

Similarly, CoA availability is closely linked to polyketide biosynthesis, as both acetyl-CoA and malonyl-CoA are synthesized as CoA thioesters and serve as essential PKS precursors. In a PKS-based DHA-producing *Y. lipolytica* strain, intracellular acetyl-CoA and malonyl-CoA pools decreased by up to 96% and 98%, respectively, during the production phase, indicating that CoA-dependent precursor depletion is a potential metabolic bottleneck [63]. To address this limitation, strategies to increase CoA availability have been explored in other yeasts. In *S. cerevisiae*, overexpression of the complete CoA biosynthetic pathway led to a 4-fold increase in total CoA levels, while expression of a feedback-resistant mutant of pantothenate kinase [64]—the first and rate-limiting enzyme in CoA biosynthesis—resulted in a 15-fold increase [65]. Likewise, overexpression of the endogenous pantothenate kinase gene (*PTK1*) in engineered *Schizosaccharomyces pombe* increased production of 3-hydroxypropionic acid, a malonyl-CoA-derived compound, by approximately 2-fold, highlighting that CoA availability can influence malonyl-CoA-dependent biosynthesis [66]. Given the observed depletion of CoA in *Y. lipolytica*, similar strategies could substantially boost malonyl-CoA availability and support high-titer polyketide biosynthesis.

### Increasing Enzymatic Capacity

Even with improved precursor availability, insufficient enzymatic capacity often remains a major bottleneck in polyketide biosynthesis. Increasing the expression levels of PKSs and their associated tailoring enzymes is therefore critical for improving overall pathway throughput. In *Y. lipolytica*, which lacks stable high-copy plasmid systems [67], this strategy is typically implemented through multicopy chromosomal integration of codon-optimized genes driven by strong promoters. Consistent with this strategy, genomic amplification of the codon-optimized type III PKS gene (*g2ps1*) from a single copy to four copies increased TAL production from 0.5 g/L to 2.1 g/L [43].

Recent advances have further expanded the genome engineering toolbox for high-copy gene integration in *Y. lipolytica*. By exploiting highly repetitive chromosomal loci, such as the 26S rDNA array present in 100–200 copies per genome, long heterologous pathways of approximately 20 kb can be integrated and stably expressed. Using this strategy, a 12-gene naringenin production pathway incorporating precursor-enhancing modules was assembled and integrated into the genome, enabling the production of 8.3 g/L naringenin in a 5-L fed-batch fermentation. These findings suggest that sufficient pathway gene dosage and balanced expression are critical for supporting high-level polyketide production [68].

In addition to increasing gene copy number, improving the catalytic efficiency of key enzymes can substantially enhance pathway flux. For example, semi-rational engineering of chalcone synthase from *Sophora japonica* (*Sj*CHS) generated the *Sj*CHS^V196A, T132C^ variant, which increased naringenin production by 60% relative to the wild-type enzyme. Further genomic amplification of this engineered *CHS* together with chalcone isomerase (*CHI*) to four copies increased naringenin titers from 24.1 to 246.4 mg/L, representing a more than 10-fold improvement over the parental strain [69]. Similar improvements have been achieved through structural engineering of upstream enzymes that support precursor supply. In the icaritin biosynthetic pathway, truncation of the first 36 N-terminal amino acids of the prenyltransferase (*EsPT*) increased icaritin production by nearly 7-fold, likely by improving its catalytic performance. In parallel, expression of a truncated 3-hydroxy-3-methylglutaryl-CoA reductase (tHMGR) lacking the N-terminal 500 amino acids was used to enhance mevalonate pathway flux and dimethylallyl pyrophosphate (DMAPP) availability. Combined with additional pathway optimization strategies, these modifications ultimately enabled de novo icaritin production of 247 mg/L in *Y. lipolytica*, representing the highest microbial titer reported to date [70].

Another prominent strategy involves modulating the spatial organization of pathway enzymes to enhance metabolic efficiency. Fusion of sequential enzymes using peptide linkers can facilitate substrate channeling, minimize diffusion of intermediates, and reduce the accumulation of undesirable intermediates. For example, fusion of 4-coumarate:CoA ligase (4CL) and stilbene synthase (STS) through a rigid EAAAK linker improved catalytic coupling between the two enzymes, reducing p-coumaric acid accumulation by approximately 18-fold while increasing resveratrol production by 1.4-fold [52]. This engineered strain ultimately achieved 22.5 g/L resveratrol in a bioreactor—the highest microbial titer reported to date [52].

## Novel Metabolic Engineering Strategies

### Rewiring Malonyl-CoA Biosynthesis via Alternative Pathways

While classical approaches such as ACC overexpression, enhanced acetyl-CoA supply, and cofactor engineering have shown great potential for improving polyketide biosynthesis, maintaining a sufficient malonyl-CoA pool throughout the production phase remains challenging. Although these approaches are practical during exponential growth, their efficiency declines markedly during stationary or high-production phases due to reduced ATP availability and tighter endogenous regulatory control, including allosteric inhibition and post-translational modifications of key enzymes such as ACC. Consistent with this challenge, intracellular acyl-CoA and malonyl-CoA levels often decline sharply upon entry into the stationary phase, thereby limiting sustained production of malonyl-CoA-derived products [64, 71]. This limitation is particularly pronounced in complex PKS systems, which impose a substantial metabolic demand for malonyl-CoA throughout prolonged fermentation.

Indeed, in a strain engineered to produce DHA through a type I PKS, intracellular malonyl-CoA levels decreased by ~98% during the stationary production phase, despite substantial upstream pathway optimization [63]. The dramatic decline in intracellular malonyl-CoA levels during the production phase suggests that the native ACC-dependent pathway alone may be insufficient to sustain precursor availability. Consequently, alternative biosynthetic routes that bypass ATP-dependent acetyl-CoA carboxylation and endogenous regulatory constraints are actively explored. Among these, malonate assimilation and the non-carboxylative malonyl-CoA (NCM) pathway expand the repertoire of strategies for reinforcing malonyl-CoA supply and supporting high-level production of malonyl-CoA-derived compounds.

The NCM pathway converts pyruvate to malonyl-CoA through the non-canonical intermediate 3-oxopropanoate without ATP consumption or carbon loss. In this pathway, BauA transfers the amino group of β-alanine to pyruvate, generating α-alanine and 3-oxopropanoate, which is subsequently converted to malonyl-CoA by the C-terminal domain of malonyl-CoA reductase (MCR-C) catalyzing the reverse of its native reductive reaction. Notably, the BauA–MCR-C module exhibited a malonyl-CoA formation rate of 2.0 μmol·min^−1^·mg^−1^ in vitro, approximately 100-fold higher than the activities reported for native ACC enzymes, highlighting its potential as an efficient alternative route for malonyl-CoA supply [72-74]. To further improve the NCM pathway, alternative β-alanine–pyruvate transaminases were evaluated in *Corynebacterium glutamicum*. Among them, Bapat from *Pseudomonas putida* exhibited the highest activity and, when coupled with MCR-C, increased flaviolin production by 280% relative to the wild-type strain, pointing to transaminase selection as a critical parameter for optimizing NCM pathway flux [75]. Although demonstrated in bacterial hosts, these results provide useful design principles for improving NCM flux in *Y. lipolytica*. In parallel, enzyme colocalization and scaffolding strategies have been explored to functionally couple the transaminase and MCR-C modules and reduce intermediate diffusion. However, only modest improvements were observed, suggesting that factors other than intermediate transfer may also contribute to flux limitation in the NCM pathway [76]. Collectively, these studies offer mechanistic insights and engineering principles that may guide future implementation and optimization of the NCM pathway in *Y. lipolytica*.

Unlike the tightly regulated ACC enzymes, the NCM pathway relies on bacterial enzymes that function largely independently of endogenous control, providing an orthogonal route for malonyl-CoA biosynthesis. This NCM pathway also enhances cellular redox balance; for instance, its implementation increased the NADPH/NADP^+^ ratio by 59% in *E. coli* [76]. In addition to its mechanistic advantages, the NCM pathway has demonstrated its applicability in *Y. lipolytica*. When incorporated into an extensively engineered palmitoleic acid (POA)-producing strain, this strategy enabled POA accumulation to 50.62% of total fatty acids, representing a 37.7-fold improvement over the initial strain [77]. As with any heterologous pathway, however, practical constraints must also be considered. One key consideration is the stoichiometric conversion of β-alanine to α-alanine during NCM cycling, which may become limiting depending on intracellular β-alanine availability. To address this challenge, β-alanine overproduction strategies have been successfully established in *C. glutamicum*, *E. coli*, and *S. cerevisiae*, providing a valuable framework for alleviating this potential bottleneck in the NCM pathway in *Y. lipolytica*.

Most recently, retrosynthesis-guided pathway design has enabled ATP-independent malonyl-CoA biosynthesis by coupling malonyl-CoA formation to the oxidative decarboxylation and deamination of two abundant amino acids, L-glutamate and L-aspartate [78]. Conceptually aligned with other noncanonical malonyl-CoA supply strategies that proceed through malonate semialdehyde, both amino acids are funneled to malonate semialdehyde (also known as 3-oxopropanoate) and subsequently converted to malonyl-CoA via MCR-C. This route provides a thermodynamically favorable driving force relative to the canonical ATP-dependent acetyl-CoA carboxylation catalyzed by Acc1, while bypassing tight regulatory constraints associated with Acc1 activity. Moreover, the use of amino acid feedstocks—potentially derived from protein-rich industrial waste streams—offers an economically attractive solution for producing high-value polyketides from low-cost resources.

In detail, in the glutamate-derived pathway, IboH, a 2-oxoglutarate-dependent glutamate dioxygenase, converts L-glutamate and 2-oxoglutarate into 3-hydroxy-L-glutamate and succinate. The resulting 3-hydroxy-L-glutamate is then cleaved by LtaE aldolase to yield glycine and malonate semialdehyde. In the aspartate-derived pathway, aspartate decarboxylase (ADC) converts L-aspartate into β-alanine, which is subsequently oxidatively deaminated by monoamine oxidase [79] to generate malonate semialdehyde, with hydrogen peroxide (H_2_O_2_) formed as a byproduct. When implemented in *Y. lipolytica*, the glutamate- and aspartate-derived pathways increased TAL production by 8.5% and 9.8%, respectively, relative to Acc1 overexpression. Beyond improving malonyl-CoA supply, these pathways also expanded the accessible chemical space by enabling the biosynthesis of 4-hydroxy-6-hydroxyethyl-2-pyrone (HHEP), a new-to-nature polyketide generated when 3-hydroxypropionyl-CoA serves as an alternative starter unit for 2-pyrone synthase. Collectively, these amino acid-derived malonyl-CoA pathways demonstrate the potential of converting low-cost and abundant amino acids into high-value natural and non-natural polyketides [78].

**Fig. 2:**
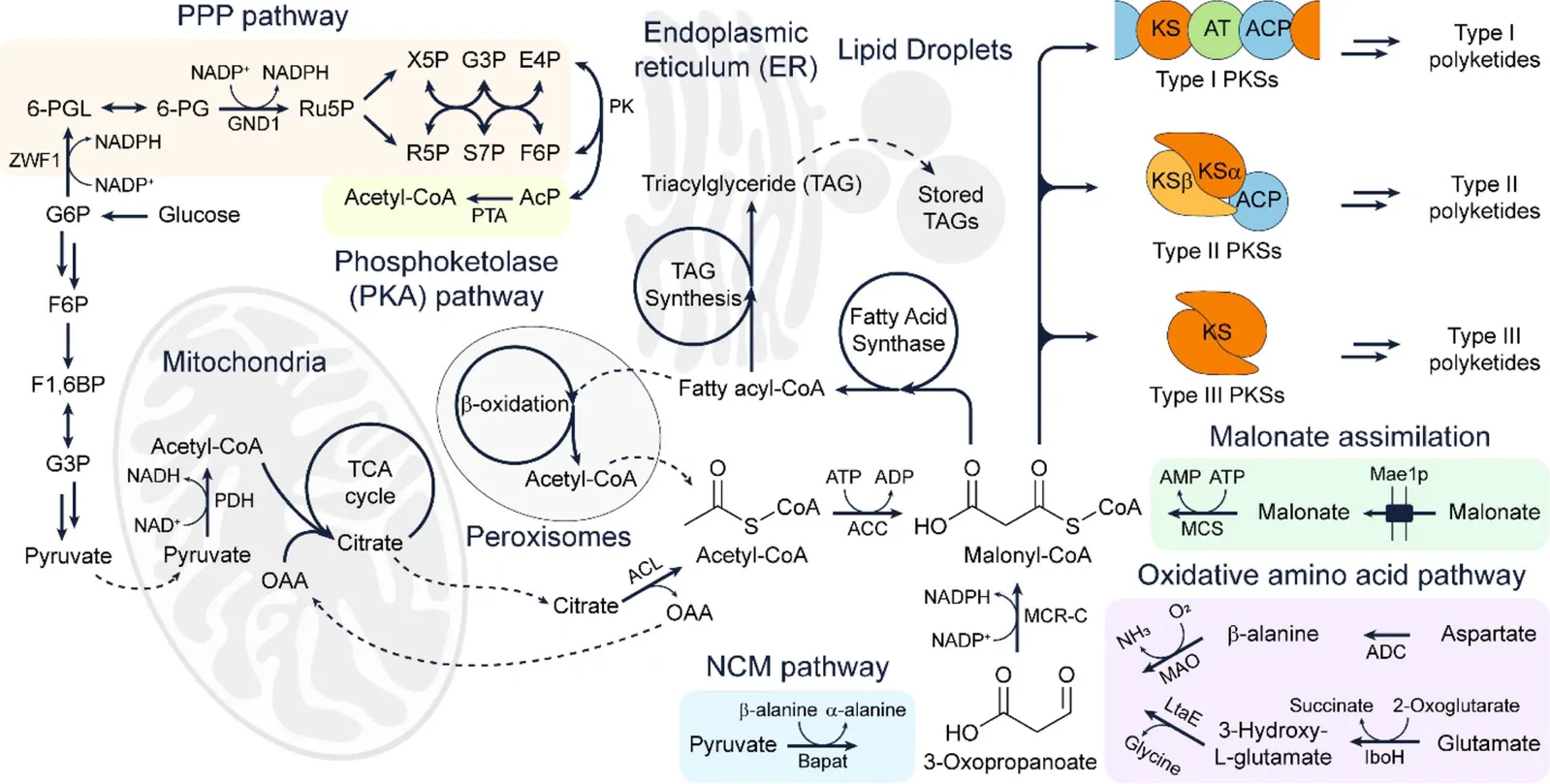
Overview of central metabolic pathways contributing to polyketide biosynthesis in *Y. lipolytica*. The pentose phosphate pathway (PPP) provides the main source of cellular NADPH. Acetyl-CoA is predominantly produced from mitochondrial citrate via ATP citrate lyase (ACL) and can also be derived from fatty acid β-oxidation in peroxisomes or through the phosphoketolase (PK) pathway. Acetyl-CoA carboxylase (ACC) converts acetyl-CoA to malonyl-CoA, which serves as a key precursor for both fatty acid synthase (FAS) and PKSs. In addition to the native ACC-dependent pathway, alternative engineered routes for malonyl-CoA production include the non-carboxylative malonyl-CoA (NCM) pathway and malonate assimilation. Triacylglycerols (TAGs) are synthesized in the endoplasmic reticulum (ER), stored in lipid droplets (LDs), and can be mobilized through peroxisomal β-oxidation to regenerate acetyl-CoA. Compartmentalization of ER, LDs, peroxisomes, and mitochondria can be leveraged to optimize spatial localization of polyketide pathways, thereby enhancing metabolic flux and overall pathway efficiency. PPP, pentose phosphate pathway; 6-PGL, 6-phosphogluconolactone; 6-PG, 6-phosphogluconate; ZWF1, glucose-6-phosphate dehydrogenase; GND1, 6-phosphogluconate dehydrogenase; Ru5P, ribulose-5-phosphate; R5P, ribose-5-phosphate; X5P, xylulose-5-phosphate; S7P, sedoheptulose-7-phosphate; G3P, glyceraldehyde-3-phosphate; F6P, fructose-6-phosphate; E4P, erythrose-4-phosphate; G6P, glucose-6-phosphate; F1,6BP, fructose-1,6-bisphosphate; PK, phosphoketolase; PTA, phosphotransacetylase; AcP, acetyl phosphate; PDH, pyruvate dehydrogenase; OAA, oxaloacetate; ACL, ATP citrate lyase; ACC, acetyl-CoA carboxylase; NCM, non-carboxylative malonyl-CoA pathway; Bapat, β-alanine–pyruvate aminotransferase; MCR-C, malonyl-CoA reductase C-terminal domain; MCS, malonyl-CoA synthetase; Mae1p, malate:proton symporter; PKS, polyketide synthase; FAS, fatty acid synthase; TAG, triacylglyceride.

In addition, another orthogonal strategy to increase malonyl-CoA availability is malonate assimilation. This approach directly converts extracellular malonate into intracellular malonyl-CoA, bypassing endogenous metabolic regulation and offering a direct route to enhance malonyl-CoA flux [80]. This pathway consists of two sequential steps: malonate import and conversion to intracellular malonyl-CoA. This modular architecture allows control over the intracellular malonyl-CoA pool by adjusting the extracellular malonate concentration (Fig. 2). Malonate assimilation has been successfully implemented and validated in several bacterial hosts engineered for polyketide synthesis, including *E. coli*, *C. glutamicum*, and *Streptomyces venezuelae*, where it has demonstrated both functionality and orthogonality to native regulatory circuits [64, 81, 82].

In contrast, the development of malonate assimilation systems in yeast has been relatively limited. Early efforts showed that heterologous expression of the *S. pombe* dicarboxylic acid transporter (Mae1) in *S. cerevisiae* enables uptake of exogenously supplied malonate [83]. Furthermore, co-expression of the *Rhizobium trifolii* malonyl-CoA synthetase (MatB) together with a heterologous malonate uptake system successfully restored growth of an ACC-bypass strain and increased its maximum growth rate by approximately 52%, demonstrating the feasibility of malonate assimilation as an alternative route for malonyl-CoA biosynthesis [84]. However, malonate assimilation remains energetically demanding, as it requires ATP-dependent activation of malonate to malonyl-CoA. In line with this energetic burden, implementation of this pathway was associated with a reduced ATP/ADP ratio during glutaric acid production [85]. Other attempts, such as balancing cellular ATP production and consumption to meet ATP demand, have been made to improve overall metabolic efficiency and redirect carbon flux toward the synthesis of ATP-dependent products [86]. For example, coordinated engineering of ATP-generating and ATP-consuming pathways has been shown to improve metabolic efficiency by maintaining cellular energy homeostasis while satisfying the ATP demand required for product biosynthesis [86]. A similar strategy could be applicable to malonate assimilation, where balancing ATP regeneration with utilization may help alleviate the energetic burden associated with ATP-dependent malonyl-CoA synthesis. Thus, although energetically costly, malonate assimilation may provide opportunities for metabolic rewiring by modulating ATP demand, potentially enabling tighter coordination between cellular energy homeostasis and target polyketide biosynthesis.

### Subcellular Compartmentalization and Organelle Engineering

Most metabolic engineering efforts have relied on the cytosol as the primary site for biosynthetic activity. However, cytosolic pathways are frequently constrained by competing side reactions and extensive metabolic crosstalk, which can limit product yields and lead to the accumulation of undesirable or toxic intermediates. To overcome the limitations of cytosolic engineering in the biosynthesis of complex natural products, subcellular compartmentalization has emerged as a powerful strategy. Unlike cytosolic engineering, subcellular compartmentalization harnesses specific intracellular structures, such as membrane-bound organelles and protein-based nanocompartments, to spatially organize entire pathways or individual reactions, thereby providing specialized microenvironments that insulate metabolic pathways from metabolic interference, enhance catalytic efficiency, and protect enzymes from proteolytic degradation [87].

*Y. lipolytica* is particularly well suited for this approach, as it contains at least 16 morphologically and functionally distinct intracellular compartments—the highest number reported among nonconventional yeasts—offering diverse platforms for spatial localization of metabolic pathways [88]. Among these organelles, peroxisomes support peroxide metabolism and fatty acid β-oxidation, supplying acetyl-CoA and NADH; the endoplasmic reticulum (ER) provides an extensive catalytic surface for membrane-associated enzymes and supports protein synthesis and folding; and lipid droplets (LDs) contribute hydrophobic microenvironments that facilitate the sequestration and transformation of hydrophobic intermediates [87].

To date, although organelles have not been exploited explicitly for polyketide biosynthesis, organelle compartmentalization has been successfully applied to the production of other acetyl-CoA-derived metabolites, particularly terpenoids [89]. Peroxisomes offer several advantages, including a high acetyl-CoA availability from fatty acid β-oxidation, as well as the ease of pathway construction enabled by relatively efficient and straightforward protein-targeting signals. For instance, peroxisomal localization of all nine enzymes involved in α-humulene biosynthesis, combined with enhanced β-oxidation to increase acetyl-CoA flux, enabled the production of 3.2 g/L α-humulene in bioreactor fermentations [90]. Similarly, peroxisomal engineering has enabled high-level squalene production, reaching 32.8 g/L through the combined strategies of β-oxidation upregulation, expression of a bacterial acetyl-CoA synthetase, and acetate co-feeding to further boost the acetyl-CoA supply [91]. Moreover, multi-organelle compartmentalization strategies have been employed to expand metabolic capacity and customize metabolic environments. In a representative study, localizing the astaxanthin biosynthetic pathway to individual organelles—such as the ER, LDs, or peroxisomes—significantly altered intermediate accumulation and increased final titers by more than 2-fold compared to cytosolic expression. Coordinated localization across all three organelles led to an even greater effect, resulting in a 4.8-fold increase in astaxanthin production [92].

Beyond providing specialized biochemical environments, organelles can also be engineered to expand their metabolic capacity and catalytic capabilities. Recent studies have focused on modulating the ER to enhance fluxes associated with polyketide and fatty acid biosynthesis. In *S. cerevisiae*, overexpression of the ER biogenesis regulator *INO2* induced ER expansion, which increased global protein synthesis and folding capacity, elevated glucose uptake, and redirected cellular metabolism toward terpenoid production through enhanced acetyl-CoA flux [93]. A similar strategy in *Y. lipolytica*, achieved via overexpression of the endogenous ER regulator *YlINO2*, promoted ER expansion and enhanced lipid biosynthesis, resulting in a 39.3% increase in lipid production [94]. However, in a taxifolin-producing *Y. lipolytica* strain, ER expansion through *INO2* overexpression or deletion of *PAH1*, a phosphatidate phosphatase that converts phosphatidic acid (PA) to diacylglycerol (DAG), did not improve taxifolin production. The authors suggested that enhanced lipid biosynthetic flux associated with *INO2* overexpression may have offset the potential benefits of ER expansion by diverting precursors away from the taxifolin biosynthetic pathway, thereby resulting in no significant increase in taxifolin production [95]. Increasing peroxisome abundance through overexpression of the biogenesis factor *PEX10* also enhanced β-oxidation capacity and thereby increased acetyl-CoA availability. This modification led to a 22% increase in TAL production, highlighting the potential of peroxisome expansion in supporting acetyl-CoA–derived polyketide biosynthesis [43].

In addition to membrane-bound organelles, alternative compartmentalization strategies have demonstrated considerable potential. Prokaryotic nanocompartments, such as bacterial encapsulins, provide a structurally defined protein-based system distinct from eukaryotic organelles. Encapsulins are hollow protein assemblies that naturally mediate selective cargo sequestration and protect labile proteins from proteolytic degradation [96]. In *Y. lipolytica*, heterologous expression of *Myxococcus xanthus* encapsulins, coupled with C-terminal targeting of PhlD—the type III PKS responsible for phloroglucinol production—enabled precise localization of the enzyme within these nanocompartments. This strategy resulted in a 120% increase in phloroglucinol titers, likely due to enhanced enzyme stability and reduced intermediate loss during its multi-step catalytic sequence [14].

Given its ability to minimize intermediate loss, concentrate pathway enzymes, and exploit organelle-specific metabolic features, organelle compartmentalization—together with broader subcellular engineering strategies—holds high promise for advancing polyketide biosynthesis in *Y. lipolytica*.

### Morphological and Cell Wall Remodeling

A significant challenge in the downstream processing of *Y. lipolytica* fermentations arises from the organism's capacity to shift from a unicellular yeast form to a filamentous morphology characterized by thickened cell walls. This dimorphic transition can be triggered by environmental stresses such as low oxygen availability, accumulation of toxic metabolites, or extreme pH conditions. Such filamentous growth not only reduces metabolic efficiency in liquid cultures but also complicates product recovery by increasing medium viscosity, impairing oxygen transfer, and reducing the efficiency of separation processes such as centrifugation and solvent extraction [97-99]. To address this challenge, efforts to modify regulatory genes and cell-wall–associated factors have suppressed filamentation while maintaining cellular growth and physiological robustness. The transcription factor Mhy1 has been identified as a key regulator of dimorphic transition in *Y. lipolytica*. Deletion of *MHY1* not only abolished filamentous growth but also improved overall cellular growth [100]. Another regulator, the kinase Cla4, similarly influences morphology, and its deletion led to reduced filament formation, albeit to a lesser extent than *MHY1* deletion [100].

These findings have supported the strategic integration of morphological engineering with metabolic pathway optimization. In one example, in the development of a strain producing scutellarin, integrating a precursor-enhancing module into the *MHY1* locus increased scutellarin titer by 151%, demonstrating the synergistic benefit of morphological suppression and metabolic pathway optimization [62]. Further advances extended this approach to the remodeling of the cell wall architecture to facilitate extractability. Genes involved in cell-wall rigidity, including those participating in chitin biosynthesis, mannoprotein assembly, and regulation of filamentous growth, were systematically deleted either individually or in combination. Among the resulting strains, three multi-deletion strains exhibited 19–33% increases in lipid production and up to 3-fold improvements in extraction efficiency, without compromising cellular growth [101].

These strategies may also enhance strain robustness under industrial fermentation conditions, and could be further complemented by engineering oxidative stress defense pathways to mitigate the accumulation of reactive oxygen species and prevent cellular damage caused by toxic metabolites [102]. Collectively, these engineering strategies suggest that morphological and cell wall engineering are effective strategies for enhancing both upstream productivity and downstream processing efficiency in *Y. lipolytica*, highlighting its potential as a next-generation biomanufacturing platform.

## Conclusion

Metabolic engineering of *Y. lipolytica* for polyketide biosynthesis has advanced rapidly, transitioning from early proof-of-concept studies to production levels approaching industrial relevance. This progress has been driven by classical strategies—such as enhancing precursor supply, redirecting carbon flux, and optimizing downstream pathways—as well as emerging approaches including subcellular compartmentalization and morphological remodeling. The distinctive physiological attributes of *Y. lipolytica*, including its high intrinsic flux toward acetyl-CoA and malonyl-CoA, robust tolerance to metabolic and environmental stress, and complex subcellular organization, collectively position this yeast as a competitive alternative to conventional microbial platforms such as *E. coli* and *S. cerevisiae*. As metabolic engineering strategies continue to mature and diversify, *Y. lipolytica* is increasingly recognized as a promising chassis for scalable and sustainable polyketide biosynthesis, with broad implications for pharmaceutical, agricultural, and specialty chemical sectors, offering a viable solution to reduce dependence on plant extraction and petrochemical synthesis routes. To date, *Y. lipolytica* has been successfully engineered to produce a diverse range of type I and type III polyketides, including 6-methylsalicylic acid, TAL, and naringenin, demonstrating its versatility as a chassis for polyketide biosynthesis [101, 103, 104].

Classical metabolic engineering approaches have established a solid basis for polyketide production in *Y. lipolytica*. Systematic enhancement of precursor supply through acetyl-CoA pathway rewiring, overexpression or deregulation of ACC, and optimization of redox and cofactor balance have consistently demonstrated the importance of fine-tuning upstream metabolic flux. Concurrently, redirecting carbon flux away from competing lipid and storage pathways, such as TAG, phospholipid, and glycogen biosynthesis, has significantly boosted polyketide production without compromising essential cellular functions. These efforts, coupled with downstream pathway optimization via multicopy chromosomal integration and enzyme engineering, have further enhanced pathway capacity. Notably, these “push–block–pull” strategies have enabled the production of resveratrol at titers of up to 22.5 g/L, the highest reported to date in microbial hosts [52] (Table 2).

In parallel, emerging metabolic engineering strategies continue to further expand the *Y. lipolytica* toolbox. Alternative malonyl-CoA biosynthesis routes, including the NCM pathway and malonate assimilation, provide distinct advantages over the native ACC-dependent pathway by reducing energy demand or bypassing tightly regulated native control mechanisms [75, 76, 84, 105]. Moreover, subcellular compartmentalization and organelle engineering utilize the inherent spatial organization of eukaryotes to enhance pathway efficiency, reduce metabolic crosstalk, and concentrate enzymatic activity. While these strategies have yet to be fully applied to polyketide biosynthesis in *Y. lipolytica*, their demonstrated success in terpenoid production and a 120% improvement in phloroglucinol titers using bacterial encapsulin-based compartmentalization demonstrate their strong translational potential [14]. Further, engineering organelle morphology and metabolic capacity provides an additional layer of metabolic control that could synergize with compartmentalized pathways [87, 93].

To enhance the industrial applicability, recent advances in morphological engineering have focused on optimizing cellular architectures to support metabolic efficiency and facilitate product recovery—two key factors for industrial scalability [50, 106]. Suppression of hyphal growth and remodeling of cell wall architecture have improved product recovery while maintaining physiological robustness. Although many of these strategies are still at an early stage of implementation in *Y. lipolytica*, their success in other microbial systems suggests considerable potential for future application.

To realize the industrial potential of these approaches, future engineering efforts should also strengthen the ability of *Y. lipolytica* to withstand the multifaceted stresses encountered during large-scale fermentation. Achieving this goal will require reinforcing host environmental robustness alongside pathway optimization. For instance, engineering endogenous molecular chaperones or heat-shock proteins could improve thermotolerance, while reinforcing oxidative stress defense systems could mitigate oxidative stress. In addition, enhancing efflux-mediated product export together with membrane lipid remodeling could alleviate the toxicity associated with hydrophobic polyketide intermediates and end products. Integrating these complementary stress-tolerance strategies with metabolic and morphological engineering is expected to further improve strain robustness and support stable, high-level polyketide production under industrial fermentation conditions.

Despite these advances, several challenges and opportunities remain. Complex PKS systems—especially types I and II—are still underexplored in *Y. lipolytica*, and the dynamic regulatory mechanisms governing precursor and cofactor control during production remain poorly understood. Addressing these limitations will require coordinated advances in large-fragment genome engineering, the heterologous expression of multi-domain PKSs, organelle-based compartmentalization, and the dynamic control of precursor and cofactor supply. Together, these strategies could improve the stable integration and functional expression of large biosynthetic gene clusters while facilitating synergistic pathway operation. In particular, organelle-based compartmentalization may spatially organize pathway enzymes and precursors to minimize intermediate diffusion and metabolic cross-talk. In parallel, dynamic regulatory systems that decouple cell growth from production can better accommodate the substantial metabolic demand imposed by complex type I and type II PKS pathways [33, 101, 107]. Looking ahead, the integration of multi-dimensional engineering strategies—metabolic, spatial, and morphological—along with emerging technologies such as machine learning, synthetic biology automation, and dynamic pathway control will likely define the next phase of polyketide biosynthesis. With continued convergence of classical and emerging toolsets, rational engineering of *Y. lipolytica* will open new avenues for sustainable, high-value production of structurally diverse polyketides.

## Figures and Tables

**Table 1:** **Comparison of *Yarrowia lipolytica* with conventional microbial hosts for polyketide production.** Performance levels are qualitatively assessed as high (***), medium (**), or low (*).

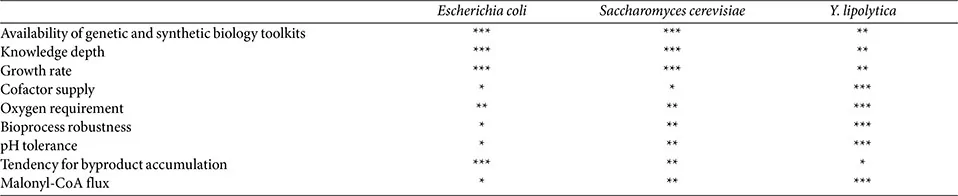

**Table 2:** Genetic engineering strategies for production of malonyl-CoA–derived polyketide products in *Y. lipolytica*.